# Effect of Amines on MIL-101(Cr) for Simultaneous H_2_S/CO_2_ Removal from Biogas

**DOI:** 10.1021/acs.energyfuels.5c01158

**Published:** 2025-05-01

**Authors:** Chunyi Li, MinGyu Song, Ryan P. Lively

**Affiliations:** School of Chemical & Biomolecular Engineering, Georgia Institute of Technology, Atlanta, Georgia 30332, United States

## Abstract

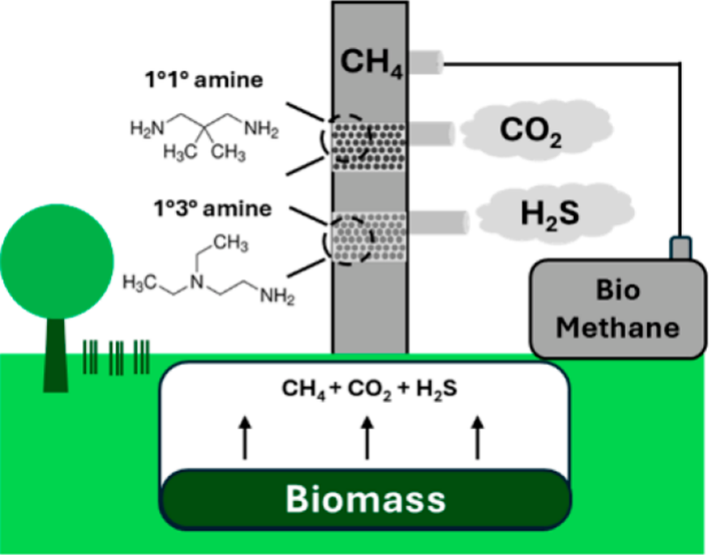

Production of biomethane from raw biogas requires the removal of impurities, such as CO_2_ and H_2_S. To accommodate the variety of biogas sources, a modular separation design with a small footprint is desired. In this work, we report three diamine-impregnated metal–organic frameworks (MOFs), MIL-101(Cr) (diamines: diethylethylenediamine, ee-2; 2,2-dimethyl-1,3-propanediamine, dmpn; *N*-methylethylenediamine, m-2) for simultaneous removal of H_2_S and CO_2_ from simulated binary and ternary biogas mixtures. The H_2_S and CO_2_ separation performance and regeneration conditions were investigated by dynamic column breakthrough and temperature-programmed desorption experiments. While all three diamine-impregnated MIL-101(Cr) samples were H_2_S-selective over CO_2_ in the mixture compositions studied, MIL-101(Cr)-ee-2 displayed the highest H_2_S/CO_2_ adsorption selectivity of ∼7. Also, it showed distinct desorption temperatures for H_2_S and CO_2_ that allows for concentrated H_2_S collection during adsorbent regeneration, which can potentially simplify the biogas separation process to achieve concentrated biomethane and H_2_S collection using the same adsorption module.

## 1 Introduction

Biogas is a renewable energy source that is widely available around the world. The abundance of waste organic matter from landfill sites, wastewater treatment, and farm wastes provides an opportunity for biogas collection and utilization.^1^ It is an environmentally friendly fuel source with potentially reduced CO_2_ and SO_2_ emissions during combustion compared to coal.^2^ The composition of biogas is similar to natural gas, and it is considered an early-stage and renewable version of natural gas, consisting mainly of methane and impurities, such as CO_2_. Purified biogas, also known as biomethane, is methane-rich. Similar to natural gas, biomethane can be used for electricity generation, household heating, and vehicular fuel.^3−7^ Because of the widely spread use of compressed natural gas in vehicles, especially for public transportation,^8^ many existing infrastructures can be used with compressed biomethane produced from biogas. Here, one major challenge after collecting biogas lies in the purification process, which is often distributed in nature compared to fossil-based natural gas systems.

Common impurities from biogas include CO_2_, sulfur species such as H_2_S, water vapor, ammonia, hydrogen, and nitrogen.^9−11^ In particular, the removal of H_2_S and CO_2_ is critical prior to further transportation and processing as both are acid gases that cause equipment and pipeline corrosion. H_2_S is highly toxic to humans and other animals. It causes respiratory tract irritation at concentrations as low as 50 ppm (parts per million). Acute exposure above 100 ppm causes immediate central nervous system injury. Although H_2_S has a strong rotten egg odor at 0.5 ppb (parts per billion), olfactory fatigue easily occurs at continuous low-concentration exposure or high concentrations. The U.S. Occupational Safety and Health Administration (OSHA) specified the ceiling for exposure as 20 ppm.^12−14^ Although CO_2_ is more benign than H_2_S to human health, it is often present in high concentrations of 30–50 vol % in biogas, causing pipeline corrosion when in contact with water and lowering the heating value of biomethane.^10^ In the U.S., the typical requirement for pipeline transportation is a H_2_S level lower than 4 ppm and a CO_2_ level lower than 2 vol %.^15−17^

Various desulfurization methods have been studied for H_2_S removal from natural gas and biogas, including biological methods using sulfur-oxidizing bacteria to turn H_2_S into elemental sulfur,^18,19^ absorption using physical solvents such as methanol, NMP, or dimethyl ethers of polyethylene glycol,^20^ absorption using alkanolamines, adsorption using solid adsorbents, etc.^21^ Adsorption is also commonly used in CO_2_ separation from gases.^22,23^ Physical solvents or aqueous amine absorption processes often suffer from high solvent volatility, a large energy requirement for solvent recovery, or increased mass transfer limitation from high-viscosity solvents. On the other hand, adsorption has the advantage of relatively low energy requirements in cyclic separation processes as it does not require heating large amounts of liquid. In particular, solid adsorbents can circumvent these problems by eliminating the use of solvents and can be regenerated by using various swing methods. For example, pressure-swing adsorption (PSA) is often used in precombustion CO_2_ separation in pressurized gas mixtures; vacuum swing adsorption (VSA) is often used in ambient pressure gas mixtures; and temperature-swing adsorption (TSA) can be employed in solid adsorbents due to the lower specific heat capacity compared to liquids.

To effectively separate H_2_S and CO_2_ from biogas, high H_2_S and CO_2_ adsorption selectivity over CH_4_ is key, because biogas is mostly composed of CH_4_. Depending on the biogas source and adsorption process, it is also desirable to control the H_2_S/CO_2_ adsorption selectivity. For example, biogas collection and purification from small-scale household waste can benefit from a compact adsorption module that removes H_2_S and CO_2_ simultaneously. In this application, adsorbents with similar H_2_S and CO_2_ adsorption selectivities are desired. On the other hand, larger-scale agricultural or industrial biogas generators can often accommodate larger purification module. In addition, these larger-scale generators can benefit from not only biomethane production but also collection of separate streams of H_2_S and CO_2_ for ease of treatment. Therefore, the ability to tune the H_2_S and CO_2_ adsorption selectivity over CH_4_ is essential in biogas adsorbent design. To achieve these goals, a class of porous solids named metal–organic frameworks (MOFs) provides a promising platform for designing adsorbents for effective H_2_S and CO_2_ removal from biogas.

The use of MOFs for CO_2_ adsorption has had extensive research activities because of the need for point-source CO_2_ capture, such as CO_2_ removal from postcombustion flue gas. At least two main themes for designing MOFs for CO_2_ adsorption exist: (1) designing MOFs with specific pore channel size to separate CO_2_ from N_2_ or CH_4_ by kinetic separation;^24^ (2) designing MOFs with specific chemical properties to selectively interact CO_2_ based on CO_2_’s high polarizability and quadrupole moment. The latter approach includes using coordinatively unsaturated metal sites with positive charges that afford strong electrostatic interaction with the CO_2_ molecules. These interactions are favorable with high heats of adsorption at low CO_2_ partial pressures. Examples of such MOFs include M-MOF-74, M_2_(dobpdc), and HKUST-1.^25−27^ However, these metal sites also form strong interactions with H_2_O molecules, rendering the sites prone to water deactivation even under ambient air conditions. Another way to utilize the coordinatively unsaturated metal sites is by the incorporation of alkylamines by a coordination bond or physical impregnation. It is well known that primary amines form carbamate species with CO_2_ to yield high CO_2_/N_2_ and CO_2_/CH_4_ selectivities by chemisorption. Because primary amines incorporated in the MOF framework do not form covalent bonds with water, these MOFs can be used, even in gas mixtures containing moisture. Amine moieties can also be incorporated using MOF linkers containing aromatic amines such as the use of 2-aminoterephthalic acid (NH_2_–BDC). Compared to using linker-based amines, physical impregnation of alkylamine takes advantage of the large pore volume of MOFs to accommodate a higher weight fraction of amines for selective adsorption of CO_2_.^28−31^

Several MOFs with high CO_2_ adsorption performance have also been studied for H_2_S adsorption. CuBTC tethered with tertiary amine TEA (triethanolamine) showed enhanced H_2_S adsorption capacity compared to the parent MOF.^32^ Reversible H_2_S adsorption has been studied in MIL-53(Al)-TDC, MFM-300(Sc), etc.^33,34^ MIL-101(Cr) modified with fluorinated group or silter ion targeted selective adsorption of H_2_S.^35,36^

Many adsorption studies are conducted on binary mixtures such as H_2_S in N_2_, overlooking the complexities in ternary systems that include both H_2_S and CO_2_.^37^ Yet, it is crucial to study these mixtures, as adsorption behavior often deviates from pure-component studies, with CO_2_ and H_2_S can both act as Lewis acids, competing for adsorption sites. Previous research highlights the challenges in maintaining MOF stability and optimizing selectivity in H_2_S/CO_2_ mixtures, revealing varied performance across different MOFs.^32,38−50^ This work addresses these gaps by investigating both binary H_2_S/CO_2_ and ternary H_2_S/CO_2_/CH_4_ adsorption using large-pore MIL-101(Cr) impregnated with various alkylamines.

The objective of this work is to develop and evaluate a potentially effective adsorbent for the selective removal of H_2_S and CO_2_ from biogas, focusing on binary H_2_S/CO_2_ and ternary H_2_S/CO_2_/CH_4_ mixtures. By employing the mesoporous MIL-101(Cr) and incorporating small alkylamines with primary, secondary, or tertiary amine functionalities, this study aims to overcome the diffusion limitations and stability challenges associated with high-molecular-weight amines. The synthesized materials are characterized by XRD, SEM, and N_2_ physisorption measurements to confirm successful synthesis and amine impregnation. Their adsorption capacities, selectivities for H_2_S and CO_2_ over CH_4_, regeneration conditions, and cyclic adsorption stabilities are investigated under simulated biogas conditions. The findings provide critical insights into optimizing MOF-based adsorbents for efficient and scalable biogas purification, addressing the challenges of acid gas removal while retaining CH_4_ purity.

## 2 Experimental Section

### 2.1 MOF Synthesis

MIL-101(Cr) was synthesized using an HF-free hydrothermal method based on published works.^47,49,51^ Chromium(III) nitrate nonahydrate (99% Sigma-Aldrich Cr(NO_3_)_3_·9H_2_O, 800 mg) and terephthalic acid (H_2_BDC, 332 mg) and deionized (DI) water (10 mL) were added to a 45 mL Teflon liner. The mixture was sonicated briefly to yield a dark blue solution. About 5 mg of MIL-101(Cr) powder was added to the mixture as a seed for the synthesis. The Teflon liner was sealed inside a digestion vessel (Parr Instrument Company) and placed in a preheated oven at 200 °C for 12 h. Following heating, the digestion vessel was allowed to cool to room temperature before opening. The resulting solid was separated by decanting the reaction solution. The solid was washed three times with DMF (35 mL each Teflon liner) using centrifuge tubes. Then, the solids from all reactors were combined and washed by mixing with methanol (50 mL) with a magnetic stir bar in a round-bottom flask for 12 h, followed by centrifugation to remove the liquid. The methanol wash process was repeated two more times. Then, the MOF powders were air-dried in a fume hood. Prior to adsorption measurements, the MOF powders were degassed at 150 °C under a vacuum for at least 12 h.

### 2.2 Amine Impregnation of MIL-101(Cr)

The MIL-101(Cr) powders were impregnated with the following diamines using a similar method to published procedures: 2,2-dimethyl-1,3-propanediamine (dmpn), *N*-methylethylenediamine (m-2), *N*,*N*-diethylethylenediamine (ee-2), and *N*,*N*-diisopropylethylenediamine (ii-2).^47,49,50^ For each diamine impregnation, the MIL-101(Cr) powder (∼500 mg) was activated at 150 °C under 30 inHg vacuum for at least 12 h to remove the adsorbed gases and coordinated terminal water molecules. The degassed powder was dispersed in methanol (8 mL) in a scintillation vial via bath sonication for 10 min. In a separate round-bottom flask, diamine corresponding to 30 wt % of the dry MOF powder was added to ∼30 mL of methanol and mixed by a magnetic stir bar. The MOF powder suspension was added to the amine solution and stirred for 24 h at 25 °C. The methanol was removed by rotary evaporation to obtain an amine-impregnated MIL-101(Cr) powder. The synthesized powder was degassed at 110 °C under vacuum prior to N_2_ physisorption measurements and single-component CO_2_ adsorption measurements.

### 2.3 Dry Breakthrough Experiments

About 150–350 mg of powder samples were loaded into stainless steel Swagelok tubes with an outer diameter of 1/8 or 1/4-in. Quartz wool was added to both ends of the tube to keep the powder sample in place. MIL-101 (Cr) and NH_2_-MIL-101(Cr) samples were degassed at 150 °C under an inert gas purge. Diamine-impregnated MIL-101(Cr) and NH_2_-MIL-101(Cr) samples were degassed at 110 °C under an inert gas purge. Then, the MOF samples were allowed to cool to 22 °C, and the inert gas was switched to the analysis gas mixture (binary mixture: 4000 ppm of H_2_S, 30 mol % CO_2_, balance He; ternary mixture: 4000 ppm of H_2_S, 30 mol % CO_2_, 30 mol % CH_4_, balance N_2_). The effluent gas concentration was monitored using a mass spectrometer (Pfeiffer GSD 320).

### 2.4 Temperature-Programmed Desorption

The MOF samples were loaded into dynamic fixed-bed breakthrough columns, and the samples were allowed to reach equilibrium with the analysis gas mixture. After equilibrium was reached with the feed gas, the analysis gas mixture was switched to a He or N_2_ purge gas at 5–20 mL(STP)/min. At the same time, the module was heated by indirect heating using heat tape controlled by a PID controller at a 10 °C/min heating rate. The effluent gas concentration was monitored using a mass spectrometer (Pfeiffer GSD 320).

### 2.5 Thermogravimetric Cyclic Adsorption Analysis

H_2_S pure-component cyclic adsorption capacities were measured with a thermogravimetric analyzer (TA Instruments Q500). Around 15 mg of MOF powder was activated under a N_2_ flow for at least 2 h. The MIL-101(Cr) and NH_2_-MIL-101(Cr) were activated at 150 °C. The MIL-101(Cr)-ee-2, MIL-101(Cr)-ii-2, MIL-101(Cr)-dmpn, MIL-101(Cr)-m-2, and NH_2_-MIL-101(Cr)-ee-2 were activated at 100 °C. Following activation, the sample was allowed to cool to 30 °C. For the 1 mol % H_2_S/N_2_ adsorption experiment, a premixed tank of 1 mol % H_2_S/balance N_2_ (Airgas) was used with flow rates of 90 mL/min (STP). The adsorption step was carried out for 60 min, followed by desorption using 90 mL/min (STP) N_2_ at the respective activation temperatures. The study was conducted by measuring the H_2_S adsorbed amount at 30 °C using a premixed H_2_S/N_2_:1/99 mixture with a thermogravimetric analyzer. In this study, the nitrogen-adsorbed amount was assumed to be negligible at 30 °C and 1 atm. By measuring the sorbent mass increase as the H_2_S gas mixture was introduced, the H_2_S adsorbed amount was calculated. For the H_2_S adsorption at other concentrations (0.4 mol % H_2_S, 0.5 mol %, and 0.6 mol %) with a N_2_ balance, the H_2_S mixture gas was made by mixing a premixed 1 mol % H_2_S/balance N_2_ gas tank and a pure N_2_ gas tank using two mass flow controllers. The total gas flow rate was 90 mL/min(STP). The subsequent adsorption and desorption steps were identical to the 1 mol % H_2_S/N_2_ experiment.

### 2.6 Elemental Analysis

About 10 mg of each diamine-impregnated MIL-101(Cr) sample was sent to Atlantic Microlab, Inc. (Norcross, GA) for elemental analysis. The C, H, and N contents were determined by combustion methods using automatic analyzers with a detection limit of 0.3%.^52^

## 3 Results and Discussion

### 3.1 Cyclic Stability in 1 mol % H_2_S

**Figure 1:**
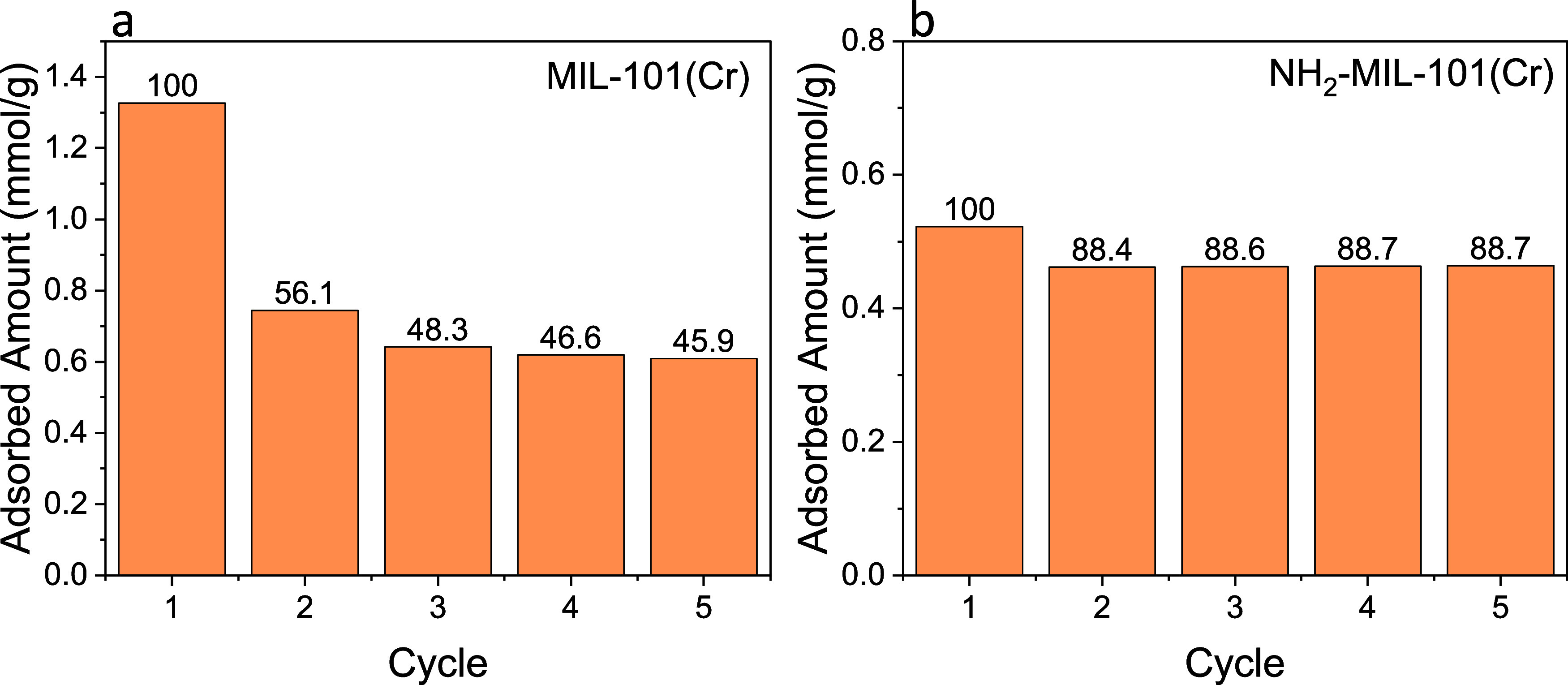
H_2_S adsorbed amount in MIL-101(Cr) (a) and NH_2_-MIL-101(Cr) (b) from five cycles of 1% H_2_S thermogravimetric analysis (TGA) using an H_2_S/N_2_:1/99 (mol/mol) mixture. MIL-101(Cr) and NH_2_-MIL-101(Cr) were regenerated under N_2_ at 150 °C after each adsorption step. The H_2_S adsorption is performed at 30 °C and 1 bar. Numbers above are the percent H_2_S adsorbed compared to the initial cycle.

The cyclic stabilities of the parent MIL-101(Cr) and amine-impregnated series of MIL-101(Cr) were investigated using 1 mol % H_2_S cyclic exposure. Figure 1 shows the amount of H_2_S adsorbed per cycle in parent MIL-101(Cr) and the NH_2_-MIL-101(Cr). The MIL-101(Cr) shows a high H_2_S uptake of ∼1.3 mmol/g in the initial adsorption cycle. However, the second adsorption cycle retained only ∼56% of the initial H_2_S adsorbed amount. The subsequent adsorption cycles showed a slow decrease in H_2_S in the amount adsorbed after each cycle. The initial sharp decrease in H_2_S adsorption capacity of ∼44% indicates that a fraction of sites (likely uncoordinated Cr metal sites) in MIL-101(Cr) adsorb H_2_S irreversibly. Between cycles 2 and 3, the fractional loss of H_2_S adsorption capacity decreased to ∼14% (from 56 to 48%). The subsequent cycles after cycle 2 exhibited a 4% decrease in H_2_S adsorption capacity. The more stabilized H_2_S adsorption capacity indicates that MIL-101(Cr) started to adsorb H_2_S reversibly after the initial two adsorption–regeneration cycles.

Shown in Figure 1b is the NH_2_-MIL-101(Cr). A decrease in the H_2_S adsorption capacity is observed between the first and second adsorption cycles. Compared to MIL-101(Cr), NH_2_-MIL-101(Cr) has a much lower adsorption capacity loss of ∼12%. Starting from the second adsorption cycle, NH_2_-MIL-101(Cr) behaves as a reversible adsorbent for H_2_S. The parent MIL-101(Cr) possessing coordinatively unsaturated Cr(III) sites is hypothesized to interact strongly with H_2_S, thereby leading to the high H_2_S adsorption capacity. Zou et al. showed that the sulfur atom on the H_2_S molecule prefers to interact with the coordinatively unsaturated Cr(III) site by physisorption.^38^ However, the observation of reversible H_2_S adsorption starting from the first adsorption cycle was not seen in this work. Possibly because of the strong Cr–O bond, MIL-101(Cr) did not experience structural degradation after H_2_S adsorption despite the strong interaction between H_2_S and MIL-101(Cr).

The role of the polar functional group in H_2_S adsorption has been studied on various MOFs including UiO-66(Zr), MIL-125(Ti), and MIL-101(Cr). Díaz-Ramírez et al. introduced fluorine group functionalized ligand for MIL-101(Cr) synthesis, resulting in 20% enhanced H_2_S uptake compared to the bare MIL-101(Cr) under 15% H_2_S/N_2_.^35^ In one study by Joshi et al., UiO-66(Zr)-NH_2_ and NH_2_-MIL-125(Ti) showed an increased breakthrough time compared to the parent UiO-66(Zr) and MIL-125(Ti).^40^ However, another study by Zou et al. found that the NH_2_ linker in UiO-66(Zr) led to irreversible H_2_S adsorption in subsequent adsorption/desorption cycles. This loss in cyclic adsorption capacity seen in UiO-66-NH_2_ is attributed to the possible formation of NH_3_HS from NH_2_ and H_2_S, which could deactivate the NH_2_ functional groups, rendering them irreversibly adsorbed to H_2_S even when regenerating at 200 °C.^38^ In the same study, MIL-101(Cr) displayed a slight enhancement in H_2_S adsorption capacity with the NH_2_ linker modification (from ∼0.49 to 0.5 mmol/g under 1% H_2_S balanced with CH_4_) in contrast to the significant decrease in H_2_S uptake with NH_2_ linker under 1% H_2_S balanced with N_2_ in Figure 1.^40^ Possible explanation is the balance between two contrary effects: enhanced selectivity toward H_2_S over CH_4_ and decreased H_2_S uptake due to the reduced pore volume by the amine group in the linker. In both cases, NH_2_ in the linker likely increases H_2_S selectivity over CH_4_ (if it exists) and decreases H_2_S uptake due to the occupation of the pore space by the amine group in the linker. Thus, in the report, H_2_S uptake of the samples was measured in the presence of a competing component (99% CH_4_), resulting in slightly higher H_2_S uptake with additional selectivity (adsorption sites) endowed by the linker amine group. On the other hand, the absence of the competitive adsorption (CH_4_) in our case showed a significant decrease in H_2_S uptake (from ∼1.3 to 0.5 mmol/g) as the comparison was conducted without any selectivity component (i.e., without CH_4_).

**Figure 2:**
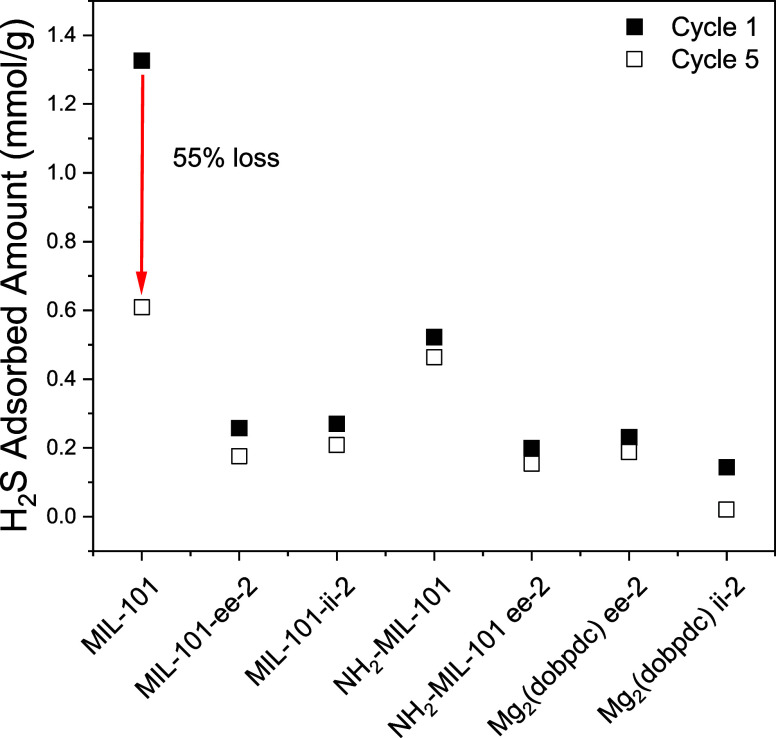
H_2_S adsorbed amount from five cycles of pure-component thermogravimetric analysis (TGA) using an H_2_S/N_2_:1/99 mixture. MIL-101(Cr), NH_2_-MIL-101(Cr), Mg_2_(dobpdc)-ee-2, and Mg_2_(dobpdc)-ii-2 were regenerated under N_2_ at 150 °C, MIL-101(Cr)-ee-2, MIL-101(Cr)-ii-2, and NH_2_-MIL-101(Cr)-ee-2 were regenerated at 110 °C in N_2_ purge following each adsorption step. The H_2_S adsorption is performed at 30 °C and 1 bar.

Due to the ambiguity in the role of the linker-based NH_2_ functional group in H_2_S adsorption capacity, as well as the absence of literature data in the cyclic adsorption capacity over multiple cycles, this study revisits the H_2_S adsorption capacities in MIL-101(Cr) and the NH_2_-MIL-101(Cr). Contrary to literature results, Figure 2 shows that NH_2_-MIL-101(Cr) has a lower H_2_S adsorption capacity than MIL-101(Cr) in all five adsorption cycles. While the NH_2_ functional group may exhibit a significant change in H_2_S interaction in the smaller-pore UiO-66 (∼11 Å),^53^ the large mesopore MIL-101(Cr) (∼29 Å) may be less sensitive to enhanced H_2_S interaction with the NH_2_ functional group, perhaps due to a lack of confinement of the H_2_S. The enhancement in H_2_S interaction with amine groups when present in a confined pore was reported in a simulation study by Zhang et al.^32^ Because of the weaker interaction between H_2_S and the NH_2_ group, NH_2_-MIL-101(Cr) showed relatively stable adsorption capacity (88.7% at fifth cycle) compared to that of MIL-101(Cr) (45.9% at fifth cycle) during cycles. For the larger pore structure of MIL-101(Cr), the electron exchange between H_2_S and Cr(III) may be more significant than the NH_2_ and H_2_S interaction; thus, the adsorption capacity of the parent MIL-101(Cr) is higher than that of the NH_2_-MIL-101(Cr). In addition, the BET surface area of NH_2_-MIL-101(Cr) is smaller than that of the parent MIL-101(Cr), which could also contribute to the decrease in H_2_S adsorption capacity.

As discussed previously, MIL-101(Cr), with its large mesopores compared to smaller-pore MOFs such as UiO-66(Zr), does not benefit as significantly from linker-based NH_2_ functionalization in terms of enhancing physisorption capacity. To better utilize its large pore structure, Lin et al. physically impregnated branched poly(ethylenimine) (PEI) in MIL-101(Cr) to target CO_2_ capture from flue gas.^54^ Later, Darunte et al. used a similar strategy for CO_2_ capture from ambient air.^47^ However, subsequent studies revealed that PEI infusion introduced significant diffusion resistance for CO_2_ molecules due to the branched nature of PEI chains, which hindered effective pore utilization. Despite MIL-101(Cr)’s large mesopores (∼29 Å), the infusion of high-molecular-weight PEI often results in ineffective distribution. In contrast, smaller-molecular-weight PEI could be infused into the MIL-101(Cr) pores instead of only coating the MOF surfaces.

To overcome the diffusion resistance caused by crowded pore environments with branched PEI, this work employs the physical impregnation of small alkylamines into MIL-101(Cr) for H_2_S and CO_2_ adsorption. Specifically, the diamines used are 2,2-dimethyl-1,3-propanediamine (dmpn), *N*-methylethylenediamine (m-2), *N*,*N*-diethylethylenediamine (ee-2), and *N*,*N*-diisopropylethylenediamine (ii-2). Additionally, ee-2 was impregnated into NH_2_-MIL-101(Cr) to explore its adsorption behavior. The smaller alkylamines circumvent the diffusional resistance associated with high-molecular-weight PEI, ensuring better accessibility of active sites within MIL-101(Cr) for H_2_S and CO_2_ adsorption.

**Figure 3:**
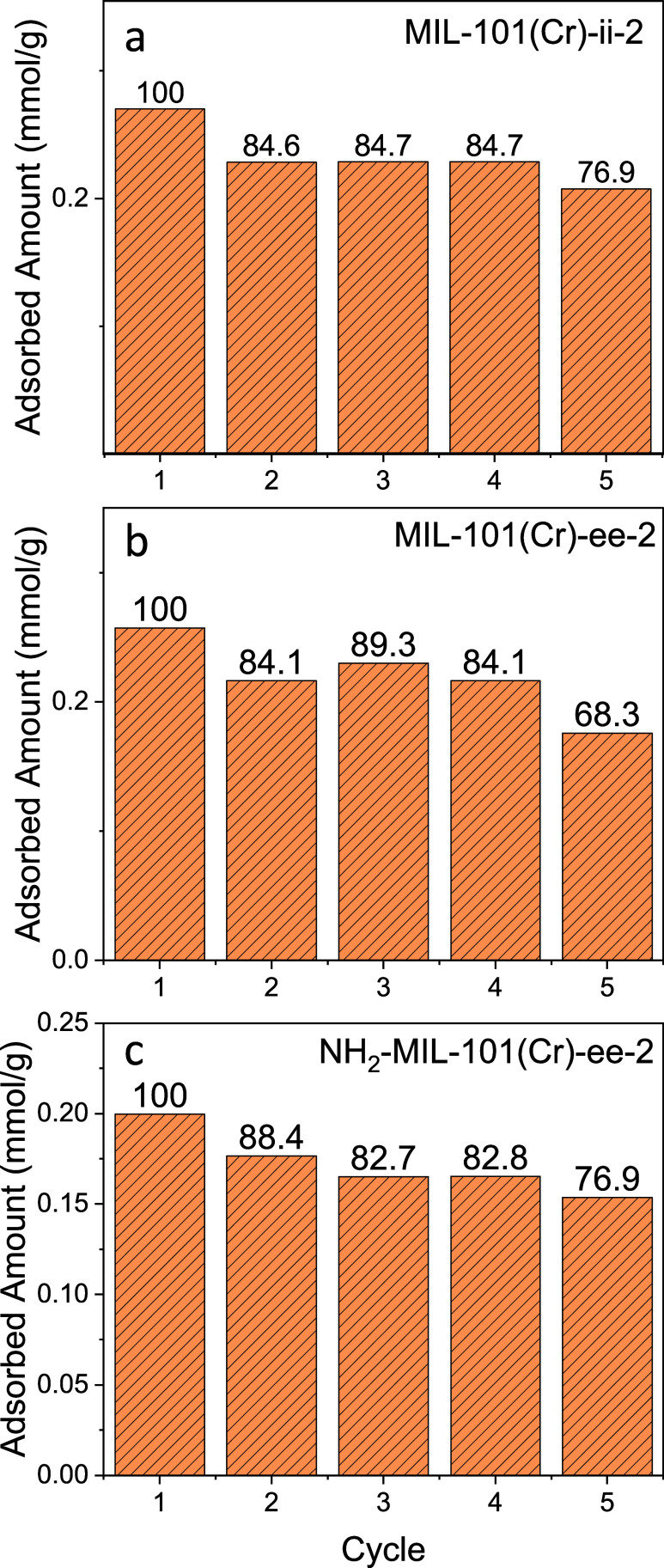
H_2_S adsorbed amount from five cycles of pure-component thermogravimetric analysis (TGA) using an H_2_S/N_2_:1/99 (mol/mol) mixture. (a) MIL-101(Cr)-ii-2, (b) MIL-101(Cr)-ee-2, and (c) NH_2_-MIL-101(Cr)-ee-2 are regenerated at 100 °C between each adsorption step. The H_2_S adsorption is performed at 30 °C and 1 bar. Numbers above bars are the percent H_2_S adsorbed compared to the initial cycle. The apparent increase in H_2_S uptake observed in the third cycle of MIL-101(Cr)-ee-2 may be attributed to an experimental error.

The same pure-component H_2_S cyclic stability test was conducted by using TGA, as shown in Figure 3. The H_2_S adsorption capacities from the initial and subsequent cycles of the amine-impregnated MOFs decreased by more than 50% compared to the parent MOFs. The decrease in capacity is possibly due to the reduction in the pore volume and BET surface area after diamine impregnation. Notably, the capacity decrease between the first and second cycles was still present in the amine-impregnated MOFs, but the percentage of capacity loss is within 16% from the first to the second cycle. The capacity decrease in amine-functionalized porous material has been reported by Okonkwo et al.^44^

The authors observed a decrease in capacity from 1 mol % H_2_S in adsorption–desorption cycles in organosilicon molecules containing primary, secondary, or tertiary amines. The strong interaction between amine and H_2_S possibly caused partial irreversibility in H_2_S adsorption cycles. In the five adsorption cycles on the amine-impregnated MIL-101(Cr) samples, the H_2_S capacity of the MIL-101(Cr)-ee-2 and MIL-101(Cr)-ii-2 is higher than the H_2_S capacity of the NH_2_-MIL-101(Cr)-ee-2. The N_2_ physisorption results indicate that the NH_2_-MIL-101(Cr) has a lower N_2_ adsorbed amount compared to MIL-101(Cr) (Figure S1), and the BET surface area of NH_2_-MIL-101(Cr) is less than half of the BET surface area of the MIL-101(Cr) (Table S1). The low H_2_S capacity of NH_2_-MIL-101(Cr)-ee-2 could also be attributed to the smaller BET surface area of the starting parent NH_2_-MIL-101(Cr) compared to that of the MIL-101(Cr).

Both amine-impregnated MIL-101(Cr)s, ee-2- and ii-2-impregnated MIL-101(Cr), the reversible H_2_S adsorption capacity is similar ∼0.2 mmol/g under 1 mol % H_2_S/N_2_ at 30 °C. Between the bulkier ii-2 and the less bulky ee-2, the similar H_2_S adsorption uptake indicates that the small change in steric hindrance in the same class of diamine does not play a significant role in H_2_S interaction strength.

Overall, the pure-component H_2_S adsorption–desorption cycles showed that amine-impregnated MIL-101(Cr) had better cyclic stability compared with the parent MIL-101(Cr). In addition, adding a linker-based NH_2_ group also increased the cyclic stability in NH_2_-MIL-101(Cr) compared to that in MIL-101(Cr).

### 3.2 H_2_S/CO_2_ Binary Adsorption Selectivity

The adsorption capacity in a binary mixture containing both H_2_S and CO_2_ was studied because the coordinatively unsaturated metal sites in MOFs and amines are commonly known to preferentially adsorb CO_2_ over N_2_ and CH_4_.^29,31^ A custom-built dynamic column breakthrough setup was used to measure the binary adsorption capacity of both H_2_S and CO_2_ in a mixture containing H_2_S/CO_2_:0.4/30 mol %, balance He. The H_2_S concentration corresponds to 4000 ppm of H_2_S, which is within the range of typical H_2_S concentrations found in biogas plants.^9^

**Figure 4:**
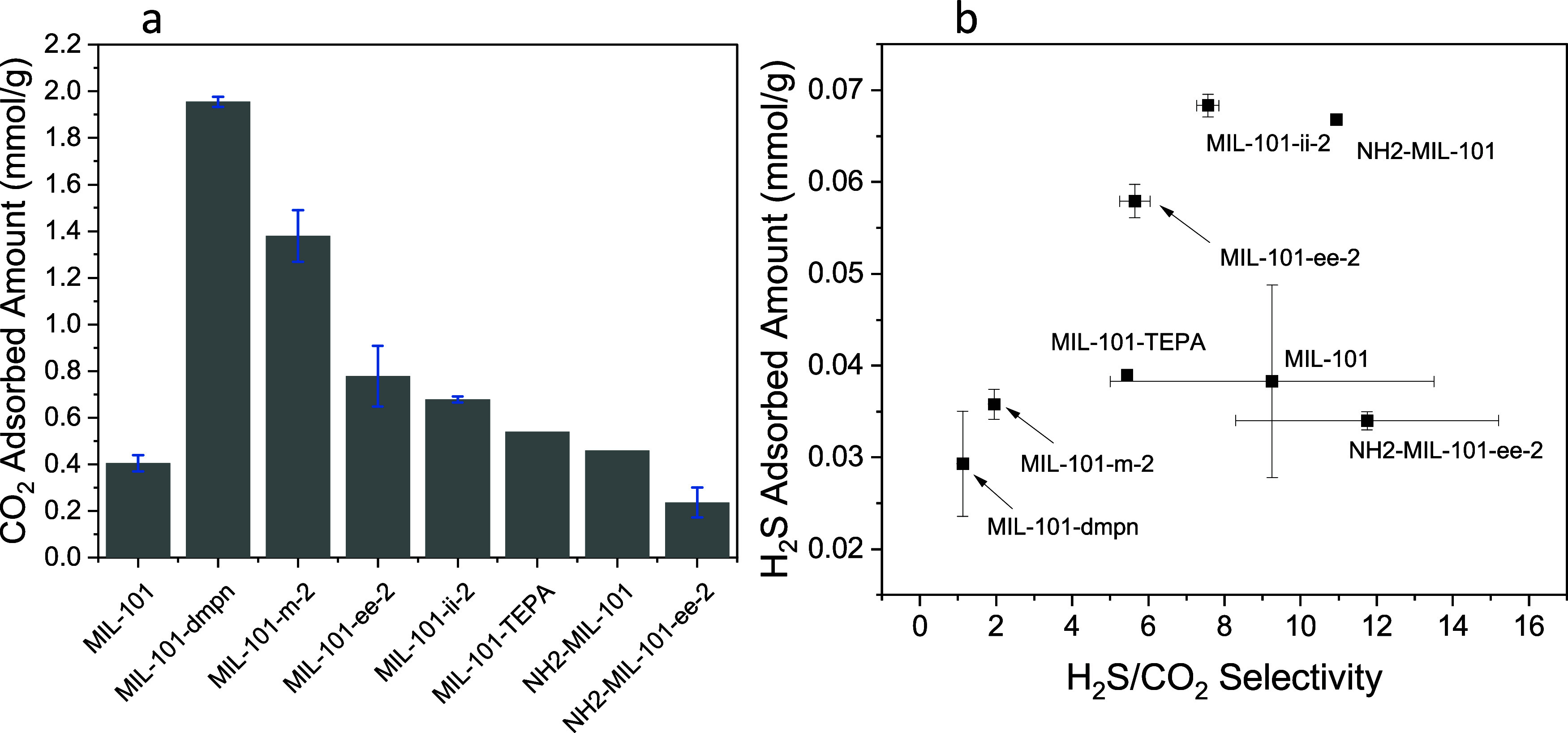
(a) CO_2_ adsorbed amount from binary mixture breakthrough experiments. (b) H_2_S adsorbed amount as a function of H_2_S/CO_2_ adsorption selectivities measured using a binary mixture. Binary mixture: 0.4 vol % H_2_S/30 vol % CO_2_/balance He. The breakthrough experiments were conducted at 295 K and 1 bar. Error bars represent the sample standard deviation from at least 2 replicate adsorption cycles, excluding the first cycle.

Figure 4 summarizes the binary adsorption capacity of a series of MOFs from the binary mixture. From the result in Section 3.1, with amine-impregnated MOFs having better cyclic stability than the parent MOFs, additional diamines are explored for postsynthesis modification of MIL-101(Cr). Besides the 1°,3° diamines, *N*,*N*-diethylethylenediamine (ee-2) and *N*,*N*-diisopropylethylenediamine (ii-2), shown in Section 3.1, two diamines containing 1°,1° and 1°,2° amines were studied. The structures of the diamines are shown in Table S2. 2,2-Dimethyl-1,3-propanediamine (dmpn) contains 1°,1° amines, and *N*-methylethylenediamine (m-2) contains 1°,2° amines.

Figure 4a reports the amount of CO_2_ adsorbed in the binary mixture. The parent MIL-101(Cr) adsorbed the least amount of CO_2_ compared to the diamine-impregnated variants. Among dmpn-, m-2-, ee-2-, and ii-2-impregnated MIL-101(Cr), the 1°,1° amine dmpn had the highest amount of CO_2_ adsorption, followed by MIL-101(Cr)-m-2, then by MIL-101(Cr)-ee-2 and MIL-101(Cr)-ii-2. It is well known that primary amines facilitate selective CO_2_ adsorption in various mixtures such as postcombustion flue gas and ambient air.^55^ The enhanced CO_2_ adsorption selectivity over N_2_ is largely contributed by the formation of carbamic acid or carbamate species between CO_2_ and primary amines. The result from this work shows that the 1°,1° amine-impregnated MIL-101(Cr) still had dominant CO_2_ adsorption capacity over the parent MIL-101(Cr), even in the binary mixture containing dilute H_2_S of 4000 ppm.

The 1°,2° amine, m-2-, impregnated MIL-101(Cr) had the second highest CO_2_ adsorption capacity among diamine-impregnated MIL-101(Cr). As expected from the pure-component CO_2_ adsorption isotherm in Figure S2, MIL-101(Cr)-m-2 has the second highest CO_2_ adsorption uptake of ∼1.10 mmol/g at 30 kPa and 299 K. Figure 4a shows that the CO_2_ adsorption uptake of MIL-101(Cr)-m-2 is unaffected by the presence of dilute H_2_S when compared to the CO_2_ uptake from isotherm in Figure S2.

MIL-101(Cr) impregnated with both 1° and 3° diamines, ee-2 and ii-2, had similar CO_2_ adsorption uptake within experimental error in the binary H_2_S/CO_2_ mixture. Between the two 1°,3° diamines, the ii-2 has bulkier isopropyl groups compared to the ethyl groups in ee-2. The more sterically hindered ii-2 did not negatively affect the CO_2_ adsorption in this study.

The NH_2_-MIL-101(Cr) and NH_2_-MIL-101(Cr)-ee-2 represent MOFs with a linker-based amino functional group. Although the pure-component H_2_S adsorption capacity was comparably high as that of MIL-101(Cr) and the cyclic stability is much better than that of MIL-101(Cr) (Figure 1), the CO_2_ adsorption capacity in the binary mixture is among the lowest compared to that of the MOFs explored in this work. Therefore, including linker-based amino functional groups in MIL-101(Cr) may not be valuable for capturing CO_2_ from dilute H_2_S-containing mixtures.

The high CO_2_ capacities in MIL-101(Cr)-dmpn and MIL-101(Cr)-m-2 are possibly a result of the high heats of adsorption between primary and secondary amines to CO_2_, and the role of the primary and secondary amines is further explored. A tetraethylenepentamine (TEPA) is impregnated into MIL-101(Cr) at a similar weight percent (wt %) as the diamines. TEPA is a longer-chain pentaamine containing both primary and secondary amine groups. However, the CO_2_ adsorption capacity in the binary mixture showed a more than 50% lower adsorption capacity compared to the MIL-101(Cr)-dmpn and MIL-101(Cr)-m-2.

The H_2_S adsorption uptakes in MIL-101(Cr) in both the pure-component case (Figure 1) and the binary mixture (Figure 2) had a significant reduction between the initial adsorption cycle and subsequent cycles. The loss in H_2_S adsorption capacity is irreversible even when the regeneration temperature reaches 150 °C. Therefore, the H_2_S adsorbed amount shown in Figure 4 is the average adsorbed amount between repeating adsorption cycles excluding the initial cycle. Compared to the diamine-impregnated MIL-101(Cr) MOFs, the parent MIL-101(Cr) has a continuous decrease in H_2_S adsorption capacity in 6 cycles. Therefore, the H_2_S adsorption capacity and H_2_S/CO_2_ selectivity standard deviations are larger compared with the diamine-impregnated MIL-101(Cr) samples.

Among the diamine-impregnated MIL-101(Cr) samples, the H_2_S adsorption capacity from the binary mixture has an opposite trend compared to the CO_2_ adsorption capacity. The 1°,1° diamine-impregnated MIL-101(Cr)-dmpn has the lowest H_2_S capacity, followed by MIL-101(Cr)-m-2. The two 1°,3° diamine-impregnated MIL-101(Cr)-ee-2 and MIL-101(Cr)-ii-2 had the highest H_2_S adsorption capacity. From these results, we hypothesize that the combination of primary and tertiary amines is optimal for simultaneous adsorption of CO_2_ and H_2_S in the binary mixture. The alkyl groups in the tertiary amine could have an inductive effect, where the electron-donating nature of the alkyl groups influences the electron density around the nitrogen, resulting in lower basicity of the diamine molecules.^56^ Therefore, the CO_2_ adsorption capacity is the lowest in the 1°,3° diamine-impregnated MIL-101(Cr). Similar to the published literature on the effect of amine types in mesoporous silica for H_2_S adsorption, the tertiary amine has increased cyclic stability over H_2_S adsorption cycles. However, in the same published study, the secondary amine has a higher H_2_S adsorption capacity than does the tertiary amine. In addition, the increased H_2_S cyclic stability is attributed to the weaker interaction between the H_2_S and tertiary amine (compared to the secondary amine),^44^ whereas this work shows that the 1°,3° diamine-impregnated MIL-101(Cr) had both increased cyclic stability and H_2_S adsorption capacity.^43,44^ The result demonstrates that the combination of primary and tertiary amines in 1°,3° diamine-impregnated MIL-101(Cr) potentially provides an optimal synergy.

Among the MOFs with the highest binary CO_2_ adsorption capacity, both ee-2- and ii-2-impregnated MIL-101(Cr) had superior binary H_2_S adsorption capacity compared with the dmpn- and m-2-impregnated MIL-101(Cr). This result suggests that 1°,3° diamine is suitable as postsynthesis modification in MIL-101(Cr) for reversible H_2_S and CO_2_ adsorption in biogas mixtures. On the other hand, MIL-101(Cr)-TEPA has relatively low CO_2_ and H_2_S adsorption capacity among the MOFs studied. Therefore, it will not be considered for further study for the subsequent biogas experiments discussed in this manuscript. It should also be noted that during the breakthrough and desorption experiments with MIL-101(Cr)-TEPA, the pressure drops in the column became too high for the mass flow controller to feed purge gas after the second adsorption cycle. Therefore, the amount adsorbed could not be obtained from the subsequent adsorption cycle.

**Figure 5:**
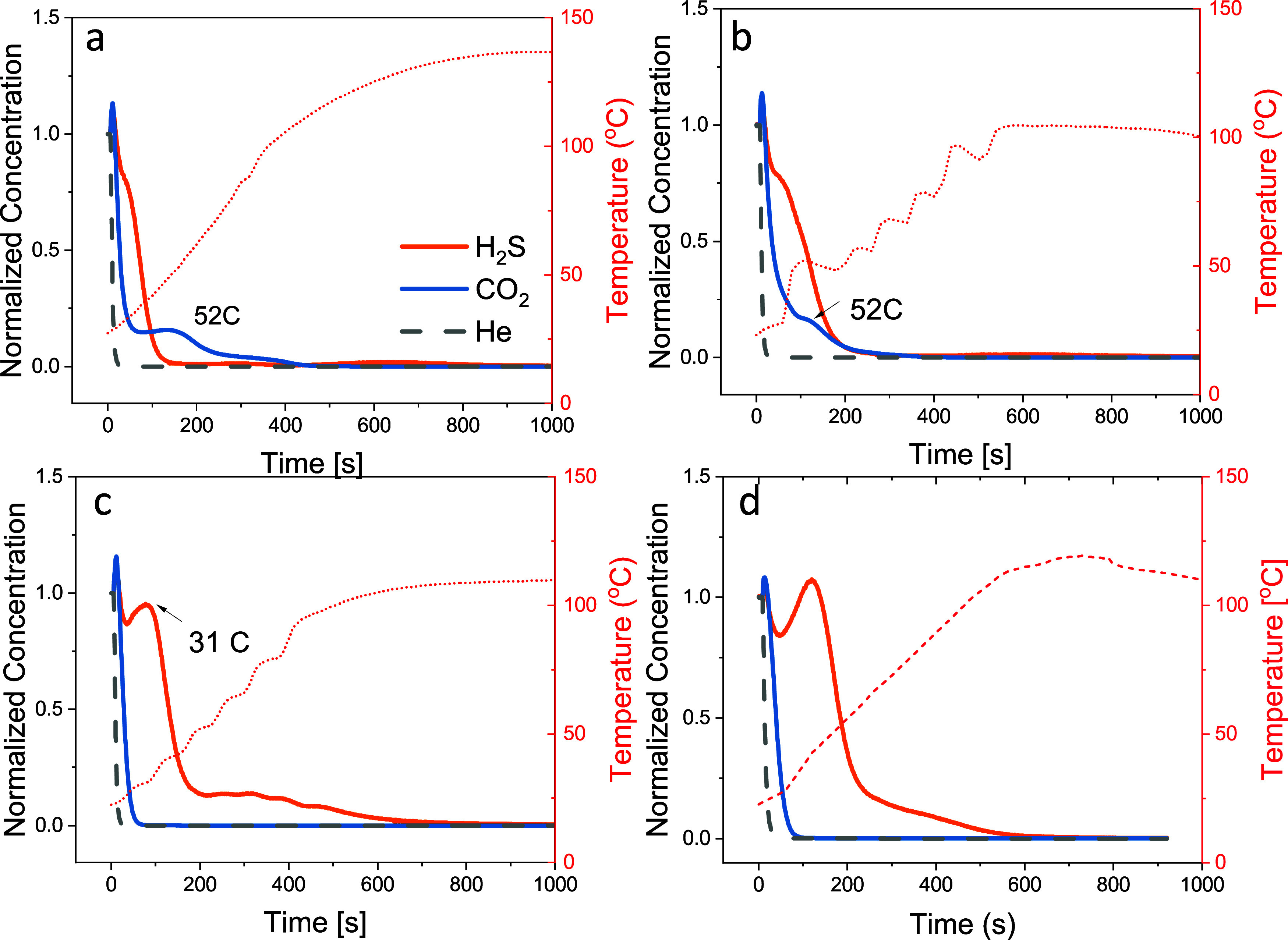
Temperature-programmed desorption following equilibrium with a mixture containing 0.4 vol % H_2_S/30 vol % CO_2_/balance He at 295 K and 1 bar. The MOFs shown are (a) MIL-101(Cr)-dmpn, (b) MIL-101(Cr)-m-2, (c) MIL-101(Cr)-ee-2, and (d) MIL-101(Cr)-ii-2. The desorptions for (a), (b), and (c) were conducted under N_2_ flow at 11 mL(STP)/min and a heating rate of 10 °C/min. The desorption for (d) was under a N_2_ flow at 7 mL(STP)/min at the same heating rate.

The NH_2_-MIL-101(Cr) was among the top-performing MOFs in terms of its pure component (Figure 3) and binary H_2_S adsorption capacity (Figure 5b). Because of the relatively low binary CO_2_ adsorption capacity compared to the diamine-impregnated MIL-101(Cr) MOFs (Figure 4a), simultaneous H_2_S and CO_2_ adsorption from biogas. However, because of its good cyclic stability in pure component H_2_S and H_2_S/CO_2_ adsorption selectivity over 11, it can be a good candidate for H_2_S separation from CO_2_-containing mixtures even at dilute H_2_S partial pressures.

It should be noted that parent NH_2_-MIL-101(Cr) and MIL-101(Cr) required regeneration temperatures of up to 150 °C between adsorption cycles. The diamine-impregnated MOFs were regenerated at 110 °C and showed reversible H_2_S adsorption in the second cycle. The coordinatively unsaturated sites in the parent MOFs offered high electron density for enhanced affinity for H_2_S. In turn, the regeneration cost also rises. This trade-off between high affinity and high regeneration cost is further explored in Section 3.1.

### 3.3 Effect of Amine in Regeneration Condition

The results from Sections 3.1 and 3.2 showed that MIL-101(Cr)-dmpn and MIL-101(Cr)-m-2 had the highest CO_2_ adsorption uptake in the pure-component isotherm and in the H_2_S/CO_2_ binary gas mixture. These two MOFs were suitable for CO_2_ adsorption in mixtures containing dilute H_2_S. For simultaneous H_2_S and CO_2_ adsorption, MIL-101(Cr)-ee-2 and MIL-101(Cr)-ii-2 had better performance in terms of their H_2_S adsorption capacity and H_2_S/CO_2_ adsorption selectivities. The effect of amine type on the regeneration condition in the MIL-101(Cr) series was studied using temperature-programmed desorption experiments. Figure 5a shows that MIL-101(Cr)-dmpn showed two distinct CO_2_ desorption peaks. The first desorption step is a sharp decrease within the first 50 s. This step represents the physisorbed amount of CO_2_ that is desorbed rapidly at low temperatures. Following the initial desorption step, a second desorption step occurs at ∼52 °C. The desorption peak at the higher temperature represents the CO_2_ adsorbed through chemisorption. Carbamic acid and carbamate species are known to form between CO_2_ and primary amines.^57^

In Figure 5b, the CO_2_ desorption profile of MIL-101(Cr)-m-2 also shows two desorption steps. The physisorbed CO_2_ typically exhibits sharper desorption peaks at lower temperatures due to the weaker interaction, while chemisorbed CO_2_ displays broader peaks at higher temperatures, indicating slower desorption kinetics and stronger adsorption. The physisorbed CO_2_ desorbs rapidly in the initial 80–100 s under ∼50 °C. Following the physisorbed CO_2_, a chemisorbed CO_2_ peak appears at a similar temperature of ∼52 °C as the MIL-101(Cr)-dmpn. Compared with the chemisorption peak in MIL-101(Cr)-dmpn, the peak in MIL-101(Cr)-m-2 is broader. The broader peak suggests potential differences in desorption kinetics. Nevertheless, distinct two-peak desorption profiles for CO_2_ were observed in both MIL-101(Cr)-dmpn and MIL-101(Cr)-m-2 samples, highlighting the roles of both physisorption and chemisorption. The rapid desorption of physisorbed CO_2_ at temperatures below 50 °C demonstrates the accessibility and reversibility of the adsorption sites, while the second chemisorption step, occurring at ∼52 °C, indicates stronger interactions between CO_2_ and the impregnated amines.In contrast to the 1°,1° and 1°,2° amine-impregnated samples, the MIL-101(Cr)-ee-2 only had one sharp desorption peak of CO_2_. This rapid desorption of CO_2_ suggests that the CO_2_ is mostly physisorbed to the MIL-101(Cr)-ee-2 sample, and it could be desorbed at low temperatures (<30 °C) under N_2_ purge.

The majority of the H_2_S desorption in MIL-101(Cr)-dmpn was completed in one step. The H_2_S desorption was slower than that of the CO_2_, and the desorption profile had a slight shoulder. The slower desorption indicated that H_2_S, although at a much lower concentration in the binary mixture, had a stronger affinity to the MOF compared to the physisorbed CO_2_. A similar H_2_S desorption profile was observed in MIL-101(Cr)-m-2 with a slight shoulder. The difference is that in MIL-101(Cr)-m-2, the H_2_S and CO_2_ desorption curves overlapped the same time span. Also, in the first 300 s of desorption, the outlet gas concentration of desorbed H_2_S was higher than CO_2_. The MIL-101(Cr)-dmpn showed a different overlap, where only the physisorbed CO_2_ desorption curve overlapped with the H_2_S desorption curve. Then, after H_2_S was almost completely desorbed, chemisorbed CO_2_ was still being desorbed from the sample. When the H_2_S and CO_2_ profiles completely overlap over the same time span, the desorption gas contains a mixture of CO_2_ and H_2_S. If concentrated H_2_S and CO_2_ are desired as products from the separation process, then this mixture requires further separation using subsequent sorbent modules or other separation techniques. MOFs with higher H_2_S/CO_2_ adsorption selectivity can potentially be targeted for collecting a concentrated H_2_S stream upon desorption. Therefore, it is desirable to select MOFs with moderate adsorption capacity for both H_2_S and CO_2_, as well as higher H_2_S/CO_2_ selectivity than MIL-101(Cr)-dmpn and MIL-101(Cr)-m-2. This approach could potentially achieve simultaneous H_2_S and CO_2_ adsorption from biogas mixtures to obtain high-concentration CH_4_ during the adsorption step. In addition, CO_2_ can be recovered via isothermal vacuum swing adsorption (VSA) and high-concentration H_2_S can be recovered via temperature-swing adsorption (TSA) during the desorption step from the same module.

With this goal in mind, the 1°,3° diamine-impregnated MIL-101(Cr)-ee-2 is selected with its third highest CO_2_ adsorption capacity among the MOFs studied in this work and an H_2_S/CO_2_ adsorption selectivity over 7. Figure 5c shows that the CO_2_ desorbed completely within the first 70 s. The black shaded area in Figure S3 represents the amount of CO_2_ desorbed, which is desorbed with the H_2_S amount desorbed until 75 s. The H_2_S desorbed amount after 75 s, shaded in orange, can be collected with high concentrations as a separate stream.

The higher affinity of the tertiary amine in ee-2 contributed to the higher H_2_S adsorption capacity in MIL-101(Cr)-ee-2. Unlike the primary and secondary amines which form covalent bonds with CO_2_, the MIL-101(Cr)-ee-2 desorbs CO_2_ under a mild temperature of ∼30 °C. The chemisorbed CO_2_ can be desorbed from MIL-101(Cr)-dmpn and MIL-101(Cr)-m-2 at ∼52 °C to achieve complete sorbent regeneration, whereas the chemisorbed H_2_S desorption in MIL-101(Cr)-ee-2 requires higher temperatures ∼75 °C. To test the hypothesis that 1°,3° diamine enhanced the H_2_S affinity while maintaining moderate CO_2_ adsorption capacity in diamine-impregnated MIL-101(Cr), another 1°,3° diamine is studied in the TPD experiments with a slightly slower purge flow rate of 7 mL(STP)/min. The MIL-101(Cr)-ii-2 has comparable CO_2_ adsorption capacity to MIL-101(Cr)-ee-2. In Figure 5d, the MIL-101(Cr)-ii-2 desorption profile showed a trend similar to that of MIL-101(Cr)-ee-2 in both CO_2_ and H_2_S desorption. The majority of H_2_S desorption occurs after complete CO_2_ desorption, making MIL-101(Cr)-ii-2 suitable for collecting concentrated H_2_S during desorption.

### 3.4 Cyclic Stability from Binary Adsorption Cycles

The powder X-ray diffraction patterns of the parent MIL-101(Cr) and post-H_2_S adsorption MIL-101(Cr) are shown in Figure S4. The PXRD patterns showed no peak loss or peak shift in the post-H_2_S adsorption sample. The results confirmed that upon H_2_S adsorption and desorption cycles, the crystallinity of the MIL-101(Cr) particles was not degraded. After adsorption–desorption cycles from the binary simulated biogas mixture, elemental analysis was used to determine the amine content in MIL-101(Cr)-dmpn, MIL-101(Cr)-m-2, MIL-101(Cr)-ee-2, and MIL-101(Cr)-ii-2 samples. The solid bars in Figure S5 represent the fraction of nitrogen remaining in each MOF sample after binary breakthrough experiments, where 100% is the total N content in the as-synthesized MOF samples. The results revealed that the nitrogen loss from all four diamine-impregnated MIL-101(Cr) samples was under 4 wt %. The negligible amine loss after H_2_S/CO_2_ mixture gas adsorption cycles suggests the recyclability of the diamine-impregnated MIL-101(Cr) samples in adsorption processes.

From this work, the types of amines did not seem to influence the cyclic stability from H_2_S/CO_2_ adsorption. Long et al. reported 1°,1° diamines to be more robust than 1°,2° and 1°,3° in Mg_2_(dobpdc) in humid SO_2_ because the amine loss was due to bisulfite formation disrupting the metal–nitrogen bond in Mg_2_(dobpdc). Unlike Mg_2_(dobpdc), most of the diamines are physically impregnated into the MIL-101(Cr) pores rather than coordinatively bonding to the metal center. Therefore, all four diamines in Figure S5 do not degrade with the same mechanism as the Mg_2_(dobpdc) (albeit in humid SO2) and show good cyclic stability.^58^

### 3.5 Effect of Amine in Ternary Mixture Adsorption

**Figure 6:**
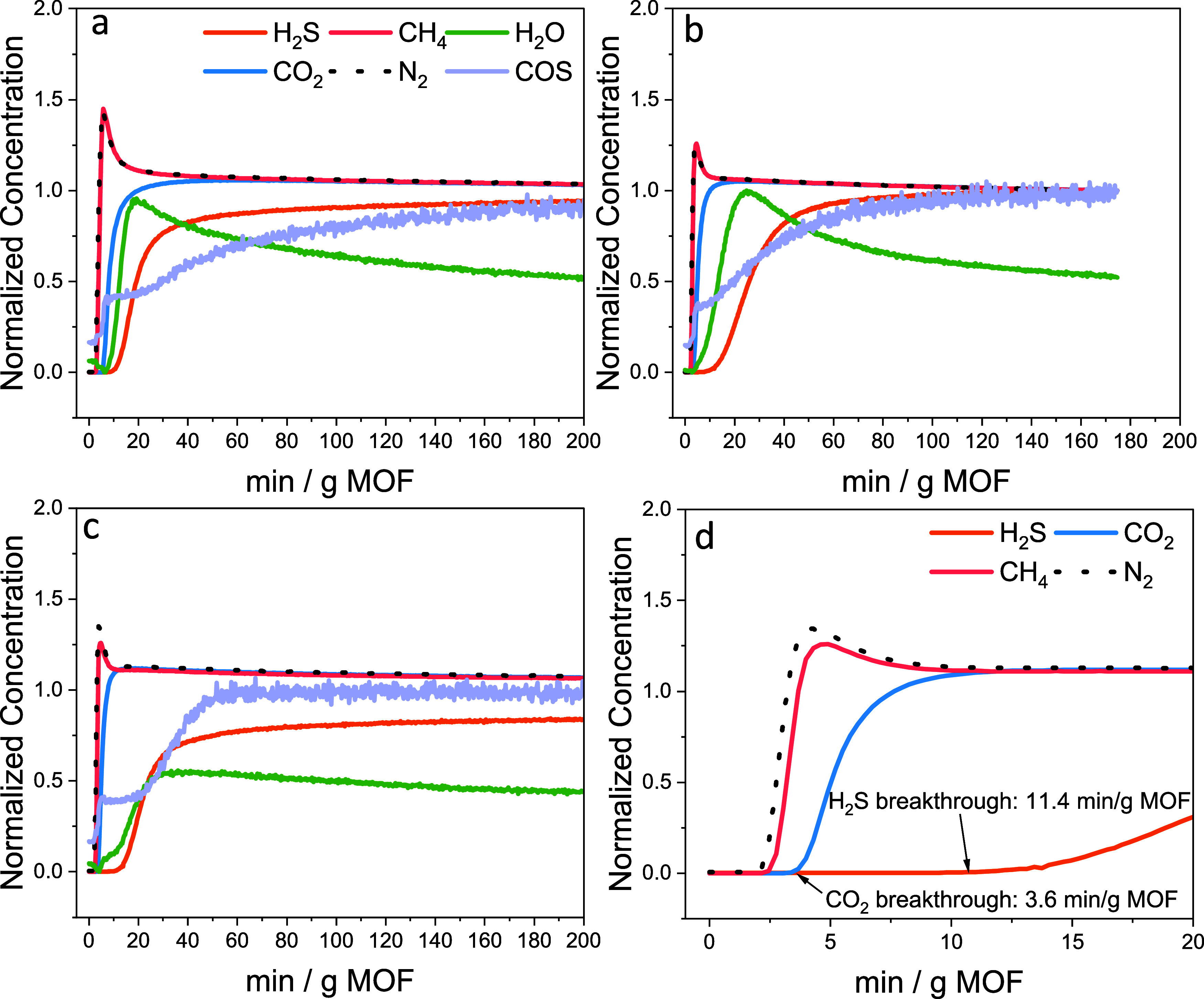
Dynamic column breakthrough experiments on (a) MIL-101(Cr)-dmpn, (b) MIL-101(Cr)-m-2, and (c, d) MIL-101(Cr)-ee-2 using 0.4 vol % H_2_S/30 vol % CO_2_/30 vol % CH_4_ balance N_2_ at 295 K and 1 bar. The gas concentrations were normalized to concentrations at equilibrium with the analyzed gas mixtures.

A simulated biogas mixture containing H_2_S/CO_2_/CH_4_:0.4/30/30 mol % with balance N_2_ was used to study the adsorption performance of the candidate MOFs selected from 3.1. Figure 6 shows the dynamic column breakthrough profiles using MIL-101(Cr)-dmpn, MIL-101(Cr)-m-2, and MIL-101(Cr)-ee-2 powder samples. In all three MOFs, CH_4_ exits the fixed-bed column immediately following the tracer N_2_ gas. This was shown by the shortest breakthrough time for CH_4_ and He. Both N_2_ and CH_4_ showed a roll-up (normalized concentration above 1), indicating the adsorbed N_2_ and CH_4_ were displaced by the incoming CO_2_ and H_2_S, as shown in the enlarged breakthrough profile of MIL-101(Cr)-ee-2 in Figure 6d. The N_2_ and CH_4_ exited the fixed-bed column ∼2.5 min after the analysis gas was fed to the sample (*t* = 0 min). Because of the low amount of N_2_ and CH_4_ adsorbed at 295 K, the delay in He and CH_4_ breakthrough indicated the void volume in the breakthrough system. In all breakthrough experiments, the ion currents from H_2_O and COS were monitored, although the exact concentration could not be determined without an internal standard. The emergence of H_2_O and COS is most likely from trace amounts of H_2_O and COS in the mixture gas cylinder. COS has been observed by other breakthrough experiments using a premixed simulated biogas mixture from Airgas.^43,59^ In MIL-101(Cr)-dmpn and MIL-101(Cr)-m-2, where the CO_2_ is more strongly chemisorbed compared to MIL-101(Cr)-ee-2, the CO_2_ showed very slow roll-up as H_2_S was adsorbed. In MIL-101(Cr)-dmpn, the CO_2_ roll-up was negligible. This result in the ternary H_2_S/CO_2_/CH_4_ mixture corresponds to the low H_2_S/CO_2_ selectivity measured using the binary H_2_S/CO_2_ mixture (Figure 4). Although the H_2_S/CO_2_ was low for MIL-101(Cr)-dmpn and MIL-101(Cr)-m-2, these MOFs were still selective toward H_2_S in the ternary mixture containing CH_4_, as shown by the delayed breakthrough time of H_2_S. The CO_2_ roll-up in MIL-101(Cr)-ee-2 was more pronounced than that in MIL-101(Cr)-dmpn and MIL-101(Cr)-m-2, suggesting that H_2_S is more strongly adsorbed and can displace some adsorbed CO_2_ from the same sites in MIL-101(Cr)-ee-2. The H_2_S/CO_2_ adsorption selectivity of MIL-101(Cr)-ee-2 was around 6.2 in the binary H_2_S/CO_2_ mixture. The results from the ternary mixture breakthrough suggest that the H_2_S and CO_2_ adsorption performance in MIL-101(Cr)-ee-2 is unaffected by the presence of 30 mol % CH_4_ in the gas mixture. Figure 6a shows that MIL-101(Cr)-dmpn has the longest breakthrough time for CO_2_, which is over 6 min. It can effectively adsorb CO_2_ and H_2_S to obtain a high-concentration stream of CH_4_ from the simulated biogas mixture. For MIL-101(Cr)-m-2 and MIL-101(Cr)-ee-2, the total adsorbed amounts of H_2_S are 0.058 and 0.036 mmol/g, respectively. MIL-101(Cr)-ee-2 is a promising candidate for simultaneous H_2_S and CO_2_ adsorption based on the ternary mixture breakthrough results in Figure 6d. The CO_2_ retention time from this study is 3.6 min/g MOF, followed by a later H_2_S retention time of 11.4 min/g MOF. During the adsorption step in a cyclic adsorption process, concentrated CH_4_ can be collected from a simulated biogas mixture before CO_2_ exits the column.

**Figure 7:**
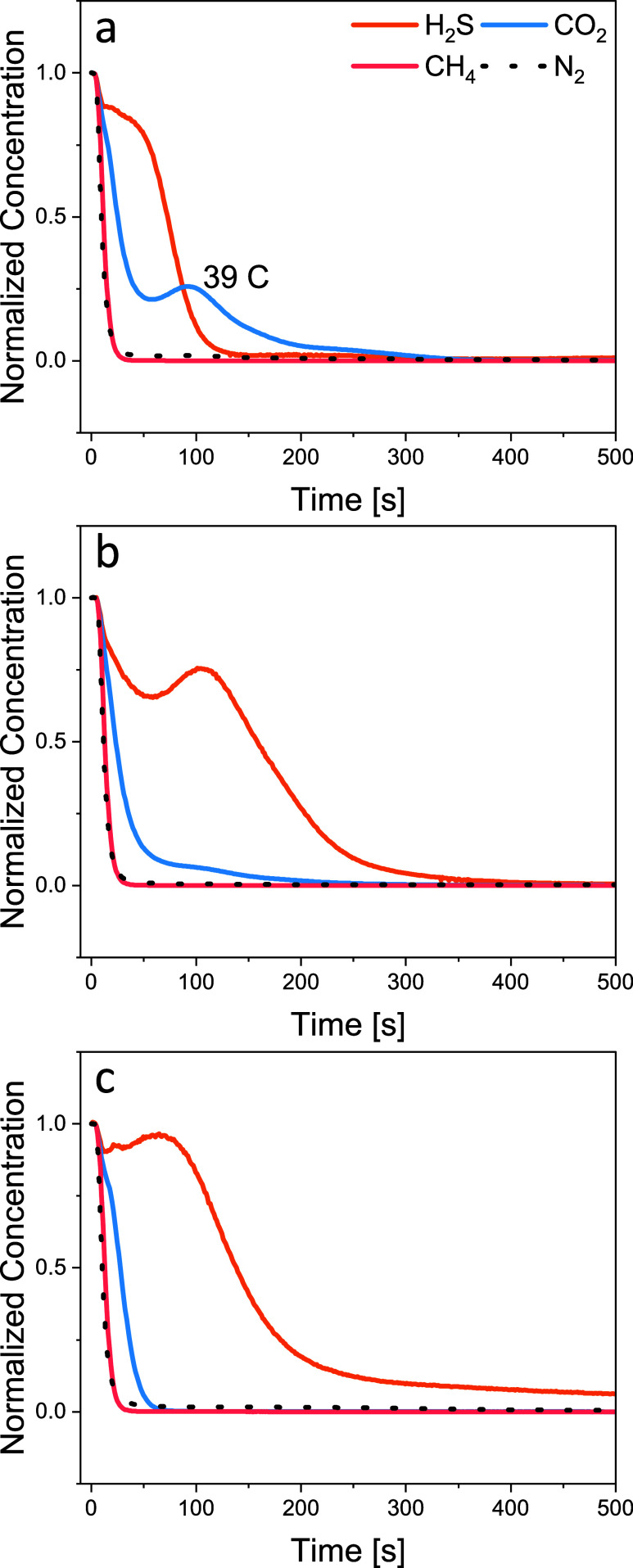
Temperature-programmed desorption following equilibrium with a mixture containing 0.4 vol % H_2_S/30 vol % CO_2_/30 vol % CH_4_ balance N_2_ at 295 K and 1 bar. The MOFs shown are (a) MIL-101(Cr)-dmpn, (b) MIL-101(Cr)-m-2, and (c) MIL-101(Cr)-ee-2. The desorption for (a), (b), and (c) was conducted under N_2_ flow at 11 mL(STP)/min and a heating rate of 10 °C/min.

The advantage of the MIL-101(Cr)-ee-2 is also shown in the desorption profile with nonoverlapping H_2_S and CO_2_ desorption discussed in Section 3.3. The TPD following the ternary mixture also showed a similar profile, as shown in Figure 7. Although MIL-101(Cr)-m-2 has a lower H_2_S adsorption capacity compared to MIL-101(Cr)-ee-2 at equilibrium, the rate of adsorption for H_2_S is much faster than the latter. In practical applications where only concentrated CH_4_ collection is concerned, the working capacity within the operating temperatures and pressures should be considered instead of the equilibrium adsorption capacities. On the other hand, if the biogas separation is operated in a temperature-swing adsorption (TSA) process, then it is more energy-efficient to operate across lower temperature spans. The MIL-101(Cr)-ee-2 showed a major H_2_S desorption peak at ∼31 °C and a second peak at ∼50 °C. The overall desorption temperature required is lower than that of MIL-101(Cr)-dmpn and MIL-101(Cr)-m-2. Therefore, MIL-101(Cr)-ee-2 could potentially require less energy during sorbent regeneration.

**Figure 8:**
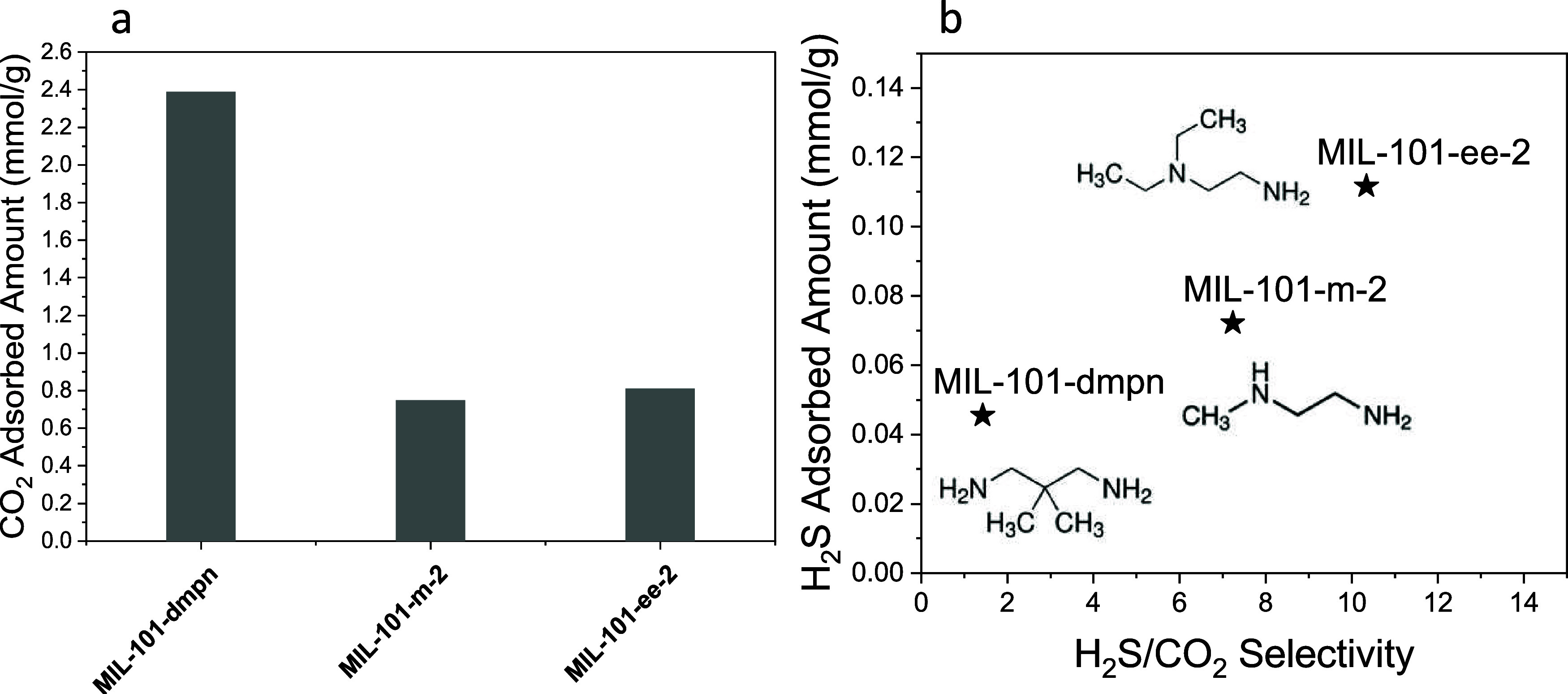
(a) CO_2_ adsorbed amount from ternary mixture breakthrough experiments. (b) H_2_S adsorbed amount as a function of H_2_S/CO_2_ adsorption selectivities measured using a ternary mixture. Ternary mixture: 0.4 vol % H_2_S/30 vol % CO_2_/30 vol % CH_4_/balance N_2_. The breakthrough experiments were conducted at 295 K and 1 bar.

The H_2_S/CO_2_ adsorption selectivity calculated from the ternary mixture is summarized in Figure 8. The MIL-101(Cr)-dmpn had the highest amount of CO_2_ adsorbed compared to the other two MOFs. The MIL-101(Cr)-m-2 adsorbed slightly less CO_2_ than MIL-101(Cr)-ee-2 from the ternary mixture. The difference in CO_2_ adsorption capacity between the binary and ternary mixtures suggests that the 1°,1° diamine retained its high uptake of CO_2_ through chemisorption, but the interaction of the CO_2_ with the 1°,2° diamine seems to be affected by the 30 mol % CH_4_ in the gas mixture. The H_2_S adsorption capacity had the same trend between the binary and ternary mixtures, where the H_2_S adsorption capacity ranked high to low from MIL-101(Cr)-ee-2 > MIL-101(Cr)-m-2 > MIL-101(Cr)-dmpn.

## 4 Conclusions

The simultaneous adsorption of H_2_S and CO_2_ from simulated binary and ternary biogas mixtures was studied using MIL-101(Cr)-ee-2, MIL-101(Cr)-dmpn, and MIL-101(Cr)-m-2. The diamine-impregnated MIL-101(Cr) samples showed more stable H_2_S adsorption capacity in adsorption/desorption cycles compared with the parent MIL-101(Cr). The effect of the amine type on H_2_S and CO_2_ adsorption was studied. The 1°1° diamine-impregnated MIL-101(Cr)-dmpn showed the highest CO_2_ adsorption capacity. The 1°,3° diamine-impregnated MIL-101(Cr)-ee-2 showed the highest H_2_S adsorption capacity and the highest H_2_S/CO_2_ adsorption selectivity under the biogas composition in this study. In practical applications of adsorption processes, it is essential to analyze the regeneration conditions to determine the adsorbed gas purity and the energy required for sorbent regeneration. The regeneration condition was studied using temperature-programmed desorption, and the result showed that the MIL-101(Cr)-ee-2 had distinct H_2_S and CO_2_ desorption peaks due to the weaker binding to CO_2_ from the tertiary amine. MIL-101(Cr)-ee-2 showed the potential to adsorb H_2_S and CO_2_ simultaneously to produce biomethane from biogas, as well as the possibility of collecting concentrated H_2_S during the regeneration step.

It should be noted that this study solely focused on the removal of H_2_S and CO_2_ from biogas, and the removal of other impurities, especially sulfurous compounds such as carbonyl sulfide (COS), carbon disulfide (CS_2_), and dimethyl sulfide (DMS), was not considered. These sulfur compounds are also known to poison Ni catalysts and should be removed for biomethane production. In addition, this work studies the equilibrium adsorption capacities of the adsorbents, and the adsorption kinetics was not in the scope of the study. In practical applications, powder adsorbents typically have high pressure drops and ineffective heat transfer. Therefore, powder adsorbents should be incorporated into structured sorbents for more efficient mass and heat transfer during adsorption processes.

## Supporting Information Available

Textural property table, nitrogen and CO_2_ isotherm raw data, temperature-programmed desorption profile, powder XRD patterns, and amine retention over H_2_S/CO_2_ adsorption cycles (PDF)
