# Supplementary material for: Effect of Amines on MIL-101(Cr) for Simultaneous H2S/CO2 Removal from Biogas
Source: Energy Fuels. 2025 May 1;39(19):9022–34. doi: 10.1021/acs.energyfuels.5c01158 (PMC12086858; doi:10.1021/acs.energyfuels.5c01158)
Supplement: Supplementary file 1 — ef5c01158_si_001.pdf [file ef5c01158_si_001.pdf]

Effect of amines on MIL-101(Cr) for simultaneous H_2S/CO_2 removal from biogas

Chunyi Li, MinGyu Song, Ryan P. Lively*

School of Chemical & Biomolecular Engineering, Georgia Institute of Technology, Atlanta, GA 30332

Email: ryan.lively@chbe.gatech.edu

Table S1. BET surface area and pore volume of diamine-impregnated MOFs

Parent MOF	Diamine	BET Surface Area (m^2/g)	Pore Volume (cm^3/g)
MIL-101(Cr)	-	3772	1.581
	Dmpn	1660	0.865
	m-2	292	0.21
	ee-2	1852	0.808
	ii-2	1866	1.36
NH_2-MIL-101(Cr)	-	1557	0.968
	ee-2	377	0.317

Table S2. Diamine type, name, and structure

Diamine Abbreviation, type		Diamine Name	Structure
**ee-2**	1°,3°	N,N-diethylethylenediamine	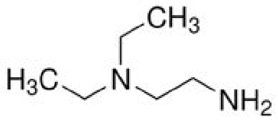
**ii-2**	1°,3°	N,N-diisopropylethylenediamine	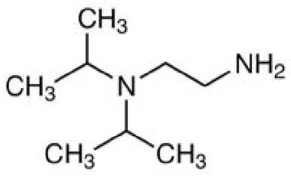
**m-2**	1°,2°	N-methylethylenediamine	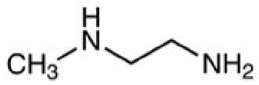
**dmpn**	1°,1°	2,2-dimethyl-1,3-propanediamine	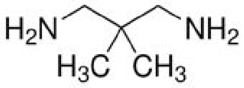

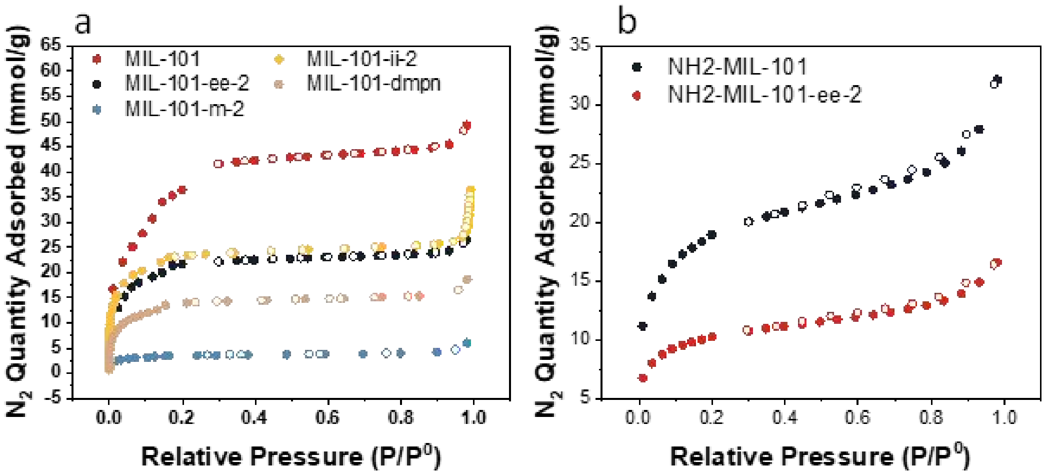

Figure S1. N_2 physisorption experiment at 77K for (a) MIL-101(Cr), MIL-101(Cr)-ee-2, MIL-101(Cr)-ii-2, MIL-101(Cr)-dmpn, MIL-101(Cr)-m-2, and (b) NH_2-MIL-101(Cr) and NH_2-MIL-101(Cr)-ee-2. MIL-101(Cr) and NH_2-MIL-101(Cr) were degassed at 150 °C under vacuum, and the diamine-impregnated samples were degassed at 110 °C under vacuum.

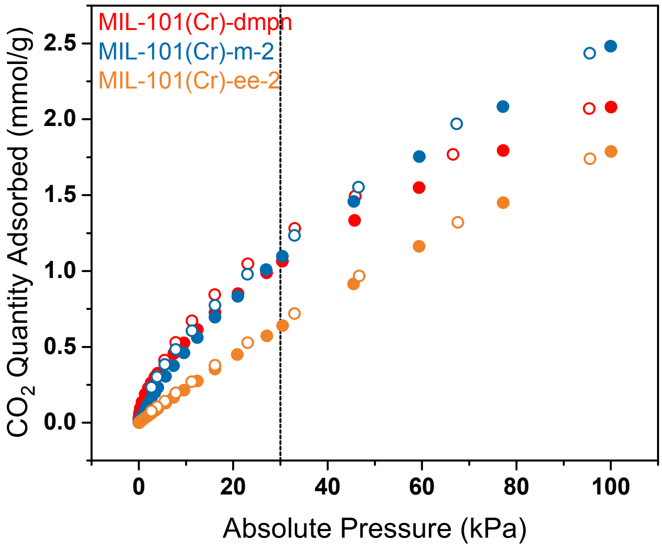

Figure S2. CO_2 adsorption isotherms of MIL-101(Cr)-dmpn, MIL-101(Cr)-m-2, and MIL-101(Cr)-ee-2 measured at 299K from 0-100 kPa. Solid circles represent the adsorption capacity during adsorption, and hollow circles represent the adsorption capacity during desorption. Vertical dashed line indicates the CO2 absolute pressure at 30 kPa.

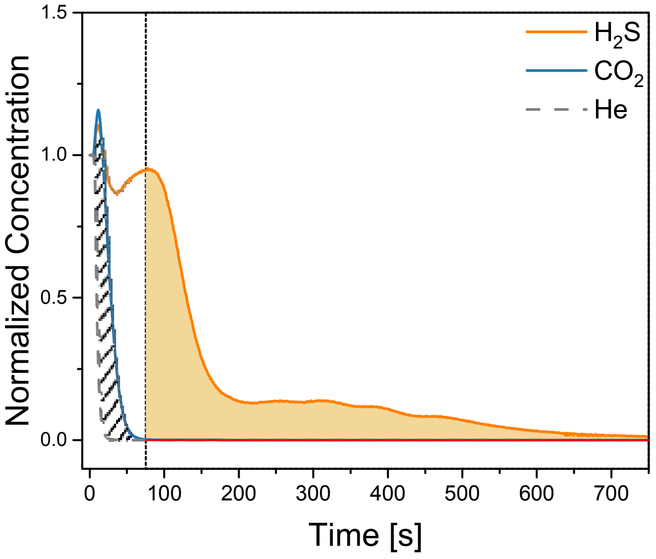

Figure S3. Temperature-programmed desorption of MIL-101(Cr)-ee-2 following equilibrium with H_2S/CO_2 0.4 vol% H_2S/ 30 vol% CO_2/balance He at 295 K and 1 bar. The desorption purge gas is N_2 at 11 mL(STP)/min. The black dash-shaded area represents the amount of CO_2 desorbed from the MOF in the first 75 seconds of desorption. The orange-shaded area represents the amount of H_2S desorbed after complete CO_2 desorption.

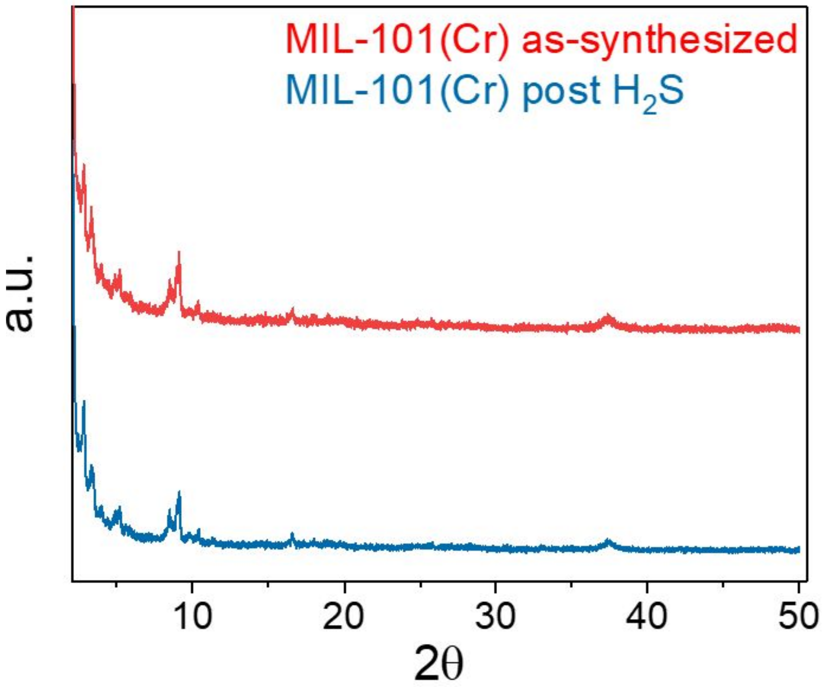

Figure S4. PXRD patterns of as-synthesized MIL-101(Cr) powder and MIL-101(Cr) powder after 1 mol%H_2S TGA experiments.

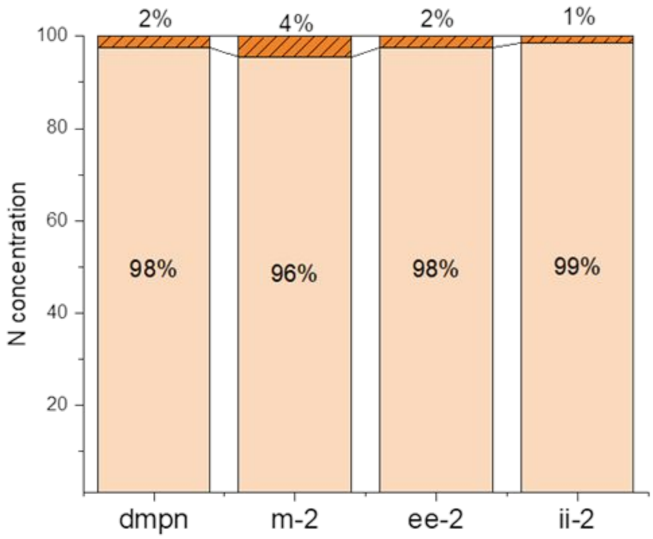

Figure S5. Amine retention in dmpn-, m-2, ee-2, and ii-2 impregnated MIL-101(Cr) after cyclic dry breakthrough experiments using H_2S/CO_2 mixture. The solid orange bar represents the percent of amine retained, and the dashed orange bar represents the amine loss after adsorption cycles
